# Utility of AI models in critical care: union of man and the machine

**DOI:** 10.1186/s13054-021-03478-9

**Published:** 2021-02-02

**Authors:** Amos Lal, Vitaly Herasevich, Ognjen Gajic

**Affiliations:** 1 Division of Pulmonary and Critical Care Medicine, Department of Medicine, Multidisciplinary Epidemiology and Translational Research in Intensive Care Group, Mayo Clinic, 200 1st St SW, Rochester, MN 55905 USA; 2 Division of Critical Care, Department of Anesthesiology and Perioperative Medicine, Multidisciplinary Epidemiology and Translational Research in Intensive Care Group, Mayo Clinic, 200 1st St SW, Rochester, MN 55905 USA

We read with great attention the remarkable Editorial by Vincent Liu highlighting the conundrums around the use of Artificial Intelligence (AI) in Critical Care [1]. Author appropriately highlighted various reasons for “cautious skepticism” around the use of this technology in the real world medicine; we wish to present a slightly more optimistic viewpoint.

We wholeheartedly agree with the author that the future AI platforms need to be conformed keeping in mind their applicability and end-user acceptance in mind. Amongst many challenges suffered by the current generation AI (Associative AI) models the 2 major issues are the “black-box” algorithms and the use of retrospective databases of EMR derivated clinical data points to refine and validate these models [2]. Due to these weaknesses the model provides prognostic information without any utility to change the outcome.

To counter these flaws, we have proposed in our preliminary work a concept of developing “Causal AI” models [3, 4]. This platform which is based on directed acyclic graphs (DAGs) developed on real life understanding of biology and pathophysiology, provides additional level of accuracy and most importantly “actionable points of intervention” which could alter the clinical course of the patient. Additionally, the transparent analytics utilized in the development of our platform provides an additional measure of trust that we hope will translate to a better acceptance by the end user, bedside clinicians and educators in this case.

Finally, we concur with utilizing the technology to “augment” our clinical decision making, cognitive training and education in the world of critical care medicine, instead of having “artificial” models that are inaccurate at best with limited clinical utility in real life. The future demands investing time and resources that will benefit our patients by improving safety and minimizing errors. Medical errors have been identified to be one of the leading causes of morbidity and mortality for our patients. Wouldn’t it be nice if we could attempt our uncertain interventions in a virtual setting using “in-silico” models before exposing our patients to any inexact mediation? Utilization of digital twins for predictive modeling has been utilized successfully for clinical decision making adjunct, research and education in chronic diseases [5]. It is imperative for this technology to be further developed and utilized in the world of critical care. Directed research is also needed to define the metrics of performance for these models and maintaining transparency of analytics in the embedded algorithms.

## Data Availability

Not applicable.
